# The Epidemic of Small-Pox in Chicago during 1877

**Published:** 1878-11

**Authors:** J. Suydam Knox

**Affiliations:** Asst. Commissioner of Health, Chicago, 1877


### THE EPIDEMIC OF SMALL-POX IN CHICAGO DURING 1877.

BY J. SUYDAM KNOX, M. D., Asst. Commissioner of Health, Chicago, 1877.

(*Remarks before the Chicago Medical Society.*)

At the present day, the opportunity to study an epidemic of small-pox, is seldom offered to the general practitioner. I would therefore submit the following propositions, as at least demonstrated in this city in the epidemic of the past year.

*I. Small-pox cannot become a serious epidemic in any city where vaccination and isolation are compulsory, and thoroughly accomplished.*

During the year 1877, in Chicago, small-pox appeared successively in five widely separated localities, viz., No. 215 West Division st., No. 555 Milwaukee ave., No. 22 Hinsch st., No. 784 Archer ave., No. 503 Blue Island ave. At each place the disease had become fully developed, several cases had occurred, and free intercommunication had been allowed. Each locality was the center of a dense population ; the people were poor and over-crowded; their sanitary conditions were inferior; many of the children were unvaccinated, and few of the adults had been vaccinated since infancy. In addition, certain occult influences (telluric or ethereal), strongly disposed to the inception of the disease. With so many favoring circumstances, an extensive and rapid spread of small-pox was to be expected, yet but 154 cases in all occurred. A surprising result in a city of 500,000 inhabitants.

I attribute this prompt suppression of the disease to the following:

Every case that could bear removal, was promptly taken to the small-pox hospital; every house that was infected, together with all its contents, was thoroughly saturated for eight hours with sulphurous acid fumes: every family within four blocks of a case of small pox, was vaccinated through house to house visitation ; in addition, every legitimate means was used to vaccinate all unprotected people throughout the city.

Lest this be considered exceptional, compare results occurring during the same year in the neighboring cities of Milwaukee and Montreal. In neither city was vaccination compulsory, nor was the removal of the sick to a small-pox hospital observed.

In Milwaukee, two per cent. of the population contracted the disease, which percentage in Chicago would represent 10,000 cases.

In Montreal, over 600 deaths from small pox occurred, which number, according to population, would represent 3,000 deaths in Chicago.

*II. Removal to hospital is to the advantage of the patient.*

The law requiring removal to a small-pox hospital, was impartially carried out in all cases, the only patients left at home, were those found in the suppurative stage of the disease.

Out of 154 cases,

116 were removed to hospital.

26 or 22.4 per cent. died.

38 were quarantined at home.

17 or 45 per cent. died.

It will be thus seen, that the chances of recovery of the patients were increased 100 per cent. by removal.

It would be absurd to assume that the simple journey to the building worked this result. It would be equally absurd to claim that it diminished it. My observation only corroborated what expert testimony long ago decided, viz., "that the removal of small-pox patients before the eighth day of the disease, works no injury to the patient.

I attribute the diminished mortality solely to the better ventilation, better sanitary surroundings, better diet, more experienced nursing, and less officious medication afforded by the hospital.

*III. Adults, vaccinated only in infancy, contract modified small-pox (varioloid) almost as readily as non-vaccinated children contract small-pox.*

In other words, they are almost as liable to the disease as if never vaccinated.

Out of 154 cases, there were: Adults, vaccinated only in infancy, 76, deaths 4; children, never vaccinated, 78, deaths, 39; average age of adults, 24 years; average age of children, 4½ years.

Infant vaccination, without re-vaccination, exerts but little influence in staying the spread of small-pox, though it wonderfully diminishes the mortality from the disease.

Herein lies the cause of epidemics of this disease. The initial small-pox patient in almost every epidemic is some adult journeying from some infected locality. The custom of vaccinating infants has become almost universal, but it is only the better classes who avail themselves of the greater protection of re-vaccination. No nation has yet framed a law by which vaccination is thoroughly and successfully accomplished.

It is an interesting question how long a primary vaccination retains its efficacy. During the past epidemic my observation was that nearly every adult re-vaccinated for the first time took the vaccine disease.

The three following cases upset the popular belief in a seven years' duration of vaccinal protection:

A. K., aged 3½ years, was successfully vaccinated by myself, although she bore the mark of a vaccination successful three years before.

E. K., mother of the above child, was successfully vaccinated by me, although she had been as successfully vaccinated three separate times in the past ten years.

Mary McG. was successfully vaccinated when three years of age. During the summer of 1877, when eight years of age, she was inspected in the public schools and passed as sufficiently protected, having a well-defined and typical vaccinal cicatrix; two weeks later she was taken down with confluent small-pox, and barely escaped with her life.

IV. *Recent successful vaccination is an absolute protection against small-pox in any form.*

Accompanying the 116 cases of small-pox sent to the hospital, were 98 relatives. These made an average stay in the building of three weeks. They were all successfully vaccinated before admission, and not a single one contracted small-pox. The families of the 38 patients left at home, were also all vaccinated, and were rigidly quarantined with the sick, yet not a case of small-pox occurred among them. The same may be said of the 20 employes of the health department who were constantly exposed to the disease.

In no instance under my observation did small-pox develop itself where primary vaccination had been successful within three years, or where successful re-vaccination had been once achieved.

V. *Successful vaccination modifies small-pox, if done during the course of the latter disease.*

This proposition is so interesting, and so generally questioned that I regret my inability to report more cases.

The following are submitted:

(1). May 12, on 19th street near Western avenue, I discovered a woman sick with small-pox, in the eighth day of the disease. In bed with her was her unvaccinated babe, aged three months. The child was immediately vaccinnated—Three days afterwards it came down with small-pox. The vaccination was retarded but successful and distinctly modified the duration of the small-pox, and the character of the eruption. The child promptly recovered.

(2). July 10th, Dr. J. M. Hall, Medical inspector, vaccinated Annie Esan, aet. six mos, at 477 N. Paulina street. At the time other members of the family in the house had small-pox.

Two days later, July 12th, small-pox showed itself in Annie. The vaccination also was successful, and the two diseases synchronously occurred in the same body. In his report of the case Dr. Hall says: "I am satisfied that the vaccination saved the child's life."

(3.) E. Wagner, aet. 18 months, living on 20th street, near Hoyne, was successfully vaccinated by Dr. E. Garrott, Medical Inspector. On the afternoon of the same day she was taken down with small-pox. The Doctor assured me that the vaccination modified the small-pox as to time and character, and that the child recovered thereby.

(4.) Mrs. Niemath, residing at 17 Broad street, was found sick with small-pox and sent to the hospital. Her son, aet. 2 years, was vaccinated, and left at home with the father. The vaccination was unsuccessful. A week afterwards, on visiting the house, the child was found just coming down with small-pox, and was again vaccinated. This second vaccination was successful, but not typical. The small-pox, however, was modified, and the child soon recovered.

(5.) S——, a babe, aet. 3 months, residing at 768 Congress street, was vaccinated by me after six days exposure to pronounced small-pox in the father. In three days the vaccine vesicles were large and well developed, when small pox appeared in the child. The whole course of the disease was that of (so-called) varioloid, there being no secondary fever to speak of, and the eruption rapidly drying up after the seventh day. The mother, who had not been vaccinated since infancy, was successfully vaccinated at the same time. The father, also vaccinated in infancy, died in hospital of hæmorrhagic small-pox.

In consulting the tables of mortality, I find that out of 24 infants under two years of age, who had never been vaccinated and contracted small-pox, 16, or 66⅔ per cent., died. If the percentage of deaths is so large, it must be more than a coincidence that the only five cases successfully vaccinated during the disease should all recover.

In reviewing my experience of the past year, I am convinced that removal to hospital should always be required, as serving the best interests both of the patients and the community; re-vaccination, as well as vaccination, should be compulsory; and vaccination should be practiced, not only as a prophylactic, but as a method of treatment of small-pox.

